# Barriers and facilitators of patient activation among older adults with cancer in China: a descriptive qualitative study based on ecological systems theory

**DOI:** 10.3389/fpubh.2026.1879945

**Published:** 2026-08-03

**Authors:** Yan Li, Xingyue Guo, Luyang Jin, Fangping Chen, Wei Kong, Yuanyuan Kuang, Shuang Hong, Yongzhen Wang, Xuemei Xian

**Affiliations:** Department of Nursing, Sir Run Run Shaw Hospital, School of Medicine, Zhejiang University, Hangzhou, China

**Keywords:** cancer, ecological systems theory, older adults, patient activation, qualitative research

## Abstract

**Background:**

Older adults with cancer face significant treatment and care challenges. Patient activation determines patients' awareness, knowledge, ability, and confidence in self-management. However, older adults with cancer generally demonstrate insufficient patient activation. This study employed a qualitative approach to investigate the multidimensional influencing factors and underlying mechanisms of patient activation among older adults with cancer, addressing the research gap concerning their subjective experiences and interactive processes from a socioecological perspective.

**Methods:**

A qualitative descriptive design was used, following the COREQ guidelines within the EQUATOR network. A purposive sample of older adults with cancer was recruited from August 15 to September 30, 2025. Data were collected through individual interviews using a semistructured guide. Theoretical and data-driven approaches were used for coding and theme generation.

**Results:**

Six themes—individual psychological strengths (microsystem), emotional empowerment through social support (mesosystem), institutional safeguards (macrosystem), individual-level functional and psychological barriers (microsystem), dysfunction within family systems (mesosystem), and barriers in health care and information services (macrosystem)—and 15 subthemes emerged from the data analysis.

**Conclusion:**

This study revealed that patient activation among older adults with cancer is shaped by multi-system factors, including patients, caregivers, medical staff, and policymakers. Coordinated interventions at each ecological system level are necessary to optimize patients' active participation in health management.

## 1 Introduction

According to the latest global health statistics report from the World Health Organization (WHO), cancer currently ranks as the first or second leading cause of death in 112 countries (1). With the intensification of global population aging, older adults have become one of the primary demographic groups affected by cancer; surveys indicate that more than 50% of patients with cancer are aged 65 years or older (2). Owing to declining physiological functions and a high prevalence of comorbidities, older adults with cancer often experience poor prognoses and diminished quality of life, posing significant challenges for treatment and care (3).

This challenge is particularly pronounced in China, where cancer and population aging are both occurring. Approximately 4.82 million new cancer cases and 2.57 million cancer-related deaths occurred in 2022, making cancer a leading cause of death in the country. This burden is compounded by rapid population aging: by the end of 2023, approximately 217 million adults were aged 65 years and older (4). Because cancer incidence increases sharply with age, older adults are disproportionately affected, with approximately 2.79 million new cases and 1.94 million deaths reported in older adults in 2022 (5). The convergence of a growing cancer burden and an aging society underscores the urgency to understand the factors that support or hinder active health management among older adults with cancer in China.

“Patient activation” is a concept largely rooted in Western healthcare cultures that emphasizes patients' awareness, knowledge, ability, and confidence in self-management, enabling them to demonstrate initiative and competence in managing their health, adhering to treatment regimens, and actively participating in medical decision-making (6). Crucially, patient activation represents the internal readiness that motivates such behaviors as self-management, treatment adherence, and active engagement in healthcare decisions, rather than these behaviors themselves. Research indicates that patients with high activation levels are generally better equipped to manage their health conditions, thereby enhancing treatment adherence and quality of life (7). Furthermore, assessing activation levels enables healthcare providers to gain deeper insights into patients' current capabilities, ensuring that any treatment or management plan is appropriately tailored to their skills, knowledge, and confidence levels, which in turn optimizes the utilization of healthcare resources (8). However, due to declining physiological functions and fear of the disease, older adults with cancer are often perceived as individuals requiring care and assistance (9). This perception gradually diminishes their motivation, knowledge, skills, and confidence in self-management. A survey conducted in the United Kingdom revealed that advanced age and a cancer diagnosis are significant contributing factors to diminished patient activation, with over 50% of older adults with cancer exhibiting low activation levels, thereby posing challenges to enhancing their engagement in self-care (10).

While patient activation has been studied extensively in Western settings, the Chinese sociocultural context presents distinctive patterns shaped by Confucian values of family harmony, filial piety, and deference to authority (11). Medical decision-making is traditionally family-centered rather than patient-driven; families may even withhold a cancer diagnosis to protect older patients from psychological distress (11). Similarly, the physician-patient relationship in China has long been characterized by a paternalistic dynamic, with patients showing strong deference to medical authority and limited engagement in shared decision-making (12). These culturally embedded norms constitute unique barriers to patient activation, underscoring the need to investigate this concept within the Chinese sociocultural context.

Patient activation levels are significantly influenced by demographic, disease-related, individual, and psychological factors, exhibiting notable variability (13). Demographic factors (14) include gender, household income, and marital status; disease-related factors (15) encompass medical history and disease stage; individual factors involve cognitive ability and health literacy; and psychological factors comprise anxiety levels and emotional state (6). However, there is a paucity of dedicated research focusing specifically on the activation levels of older adults with cancer. Most existing studies rely on quantitative designs that capture activation scores but provide limited insight into the subjective experiences, emotional processes, and interpersonal dynamics underlying these scores (16). In particular, the core dimensions of patient activation-such as confidence, capability, and motivation—and how they are shaped by the interplay between individual, family, and systemic factors remain inadequately understood in this population. Furthermore, no study to date has examined patient activation among older adults with cancer from a socio-ecological perspective that accounts for multi-level influences. Qualitative research is well suited to address this gap, as in-depth interviews can capture the lived experiences and meaning-making processes that quantitative instruments may overlook.

Accordingly, this study employed a qualitative descriptive design within the framework of ecological systems theory (EST) to systematically explore the barriers and facilitators of patient activation among older adults with cancer in China, with the aim of informing targeted, multi-level interventions to enhance their self-management capacity, treatment adherence, and quality of life, thereby reducing the burden on healthcare systems.

## 2 Materials and methods

### 2.1 Theoretical framework

This study is grounded in ecological systems theory (EST), an interdisciplinary framework that examines how individual behavior is shaped by interactions with the social environment (17). EST classifies the social environment into three interconnected systems: the microsystem (individual-level factors), the mesosystem (interpersonal and family-level dynamics), and the macrosystem (institutional, cultural, and policy-level contexts) (18). In the present study, this framework was operationalized as follows: the microsystem encompasses patients' physiological condition, psychological state, and self-efficacy; the mesosystem includes family support, informal caregiving, and peer relationships; and the macrosystem covers health care institutions, medical insurance policies, and broader sociocultural norms. The exosystem (indirect environmental influences such as caregivers' workplaces) and chronosystem (temporal changes such as policy shifts over time) were acknowledged as contextual background but not included in the analytical framework, as the study focused on ecological levels directly experienced and articulated by participants. By adopting this multilevel lens, this study systematically identifies barriers to and facilitators of patient activation and examines how factors across ecological levels interact to influence older adults' health management behaviors (19).

### 2.2 Study design

This study employed a qualitative descriptive design, which aims to provide a comprehensive, low-inference summary of participants' experiences in their own words, without imposing a preexisting theoretical interpretation. Data were analyzed using reflexive thematic analysis following the six-phase approach. This combined approach was chosen because qualitative description is well suited to exploring underinvestigated phenomena in health research, whereas thematic analysis provides a flexible and systematic procedure for identifying patterns across interview data. Semistructured individual interviews were conducted with older adults with cancer, guided by ecological systems theory, to explore factors that facilitate or hinder patient activation in self-management.

### 2.3 Setting and participants

This study was conducted at a tertiary hospital in Hangzhou, China, from August 15, 2025, to September 30, 2025. Participants were recruited from two specialized oncology departments. Purposive sampling was employed, guided by the principle of maximum variation. Participants were intentionally selected to ensure diversity across seven dimensions: sex, age, cancer type, disease stage, treatment modality, medical insurance type, and primary caregiver type. The following inclusion and exclusion criteria were applied: aged 65 years or older with a confirmed diagnosis of cancer; were undergoing consistent and stable chemotherapy or radiotherapy without surgical intervention; had tumors stage I–II; and could communicate verbally in Mandarin or the local dialect and participate in an interview without assistance; and provided informed consent and voluntarily agreed to participate in this study. Patients were excluded if they had physical conditions or illness-related fatigue that precluded participation in an interview or had cognitive impairment or a diagnosed psychiatric condition that would affect their ability to provide informed responses.

Recruitment was conducted by the principal investigator (LY) in collaboration with ward nurses, who identified eligible patients from the inpatient roster and introduced the study to those meeting the inclusion criteria. Data collection continued until thematic saturation was achieved, which was defined as the point at which no new themes or subthemes emerged from consecutive interviews (20). Saturation was assessed throughout the data collection process through iterative review of the interview transcripts. Data collection concluded after 18 interviews, at which point the research team determined that thematic saturation had been reached, as no new codes or themes had emerged from the final several interviews.

Participants were restricted to stage I–II disease to ensure that their reflections on activation were not confounded by acute treatment complications or end-of-life concerns. Single-site recruitment from a tertiary hospital and the exclusion of more vulnerable subgroups represent boundary conditions whose implications for generalizability are addressed in the Limitations section.

### 2.4 Data collection

The interview guide was developed on the basis of ecological systems theory (Table 1) and was finalized after expert review and two rounds of pilot testing. Prior to this study, the principal investigator, LY—a nurse with 2 years of clinical oncology experience and a professional master's degree in nursing—had completed formal qualitative research methodology training and supervised interview practice. LY had no prior clinical or caregiving relationship with any participant and was introduced solely as a researcher to minimize the influence of the clinician–patient dynamic. Face-to-face interviews were conducted in a quiet, private room with only the researcher and participant present; each lasted 30–45 min and was audio-recorded after written informed consent was obtained. The researcher employed open-ended probing techniques, including paraphrasing and reflective questioning, while leading questions were avoided. Non-verbal cues (facial expressions, gestures, vocal intonation) were documented during each interview and integrated into the transcripts. To address reflexivity, LY maintained a reflexive journal that recorded initial impressions, potential biases, and analytical decisions throughout the data collection; these entries were reviewed during regular team debriefing sessions. To minimize interviewer bias, in addition to the rigorous development of the interview guide and the use of open-ended probing techniques, regular team debriefing sessions were held after every three to four interviews to review interview techniques and bracket emerging preconceptions.

**Table 1:** Interview questions for older adults with cancer.

Microsystem
1. Would you tell me what changes happened in your life after being diagnosed with cancer? 2. What difficulties have you encountered in dealing with illness, and how did you overcome them? 3. What changes have you made regarding the treatment and what factors led you to make these changes? 4. Can you share your true feelings and experience of managing your health now? And if are you satisfied with your performance, why? 5. “Patient activation” refers to the competencies, capabilities, and self-confidence that an individual demonstrates in managing their own health. Which of your behaviors do you consider to be indicative of patient activation? 6. What factors do you think are barriers for you actively managing your own health care?
Mesosystem
7. How would you describe the dynamics within your family, and in what ways do their behaviors facilitate or hinder your activation? 8. What are the sources of support you perceive from individuals outside your family, and how do they specifically influence your activation?
Macrosystem
9. What is your perspective on the impact of the current social environment, including health care systems, policies, and cultural contexts, on the autonomous disease management practices of the elderly population? 10. From your perspective, what social-cultural and policy safeguards would you consider essential to facilitate the activation of your disease management?

### 2.5 Data analysis

Within 24 h of each interview, audio recordings were transcribed verbatim, with non-verbal cues (facial expressions, gestures, pauses) annotated at corresponding points and participant demographic information appended. The analytic procedure followed reflexive thematic analysis and integrated deductive and inductive coding in three phases. In Phase 1 (familiarization and initial coding), the first author read and reread all the transcripts to develop immersion in the data. An initial coding framework was then constructed based on the three EST levels—the microsystem, the mesosystem, and the macrosystem. In Phase 2 (hybrid coding), each transcript was coded using two parallel processes. Deductively, text segments were mapped onto the three EST levels on the basis of the theoretical framework. Inductively, the first author simultaneously generated data-driven codes within and across the EST levels, allowing subthemes to emerge organically from participants' accounts (e.g., “treatment-induced physical decline” emerged inductively within the microsystem). When inductive codes could not be accommodated within an existing EST level, the research team discussed whether to refine the level's scope or create a new subcategory, thereby ensuring that the theoretical structure remained responsive to the data. In Phase 3 (theme development and verification), the preliminary themes and subthemes were reviewed for internal coherence and mutual distinctiveness. For conceptually adjacent themes—such as positive illness appraisal (cognitive reframing of the diagnosis), self-efficacy (confidence in one's capacity to act), and engagement in health management (observable behavioral change)—the team distinguished them analytically by their functional locus: cognitive appraisal, perceived capability, and behavioral enactment, respectively (Table 2). A coding manual was developed, and a second researcher independently coded 30% of the transcripts using this manual. Discrepancies were resolved through discussion and, where necessary, consultation with the original interview data. Member checking was conducted by returning preliminary findings to a subset of participants to verify the interpretive accuracy. Consistent with the reflexive thematic analysis approach, inter-rater reliability statistics such as Cohen's kappa were not computed, as such measures assume independent coding and do not capture the collaborative meaning-making process central to reflexive analysis (21). Instead, the dual-coding process served to enrich interpretive depth and challenge analytical assumptions through discussion and consensus.

**Table 2:** Example of the coding process.

Transcript	Codes	Subthemes	Themes
“At my age, falling ill is natural. Compared to the hardships I endured in my youth, this is just a minor setback. I will calmly undergo treatment without worrying too much.” (P6)	Falling ill is natural	Positive illness appraisal	Individual psychological strengths (microsystem)
	Compared to the hardship before		
	A minor setback		
	Calmly undergo treatment		
“I have also seen younger people around me lose their lives to cancer or accidents. Compared to them, I am already lucky to have a chance at getting treatment.” (P10)	Compared to younger people who lose lives to cancer or accidents		
	Lucky to have a chance at getting treatment		
“Previously, I have always enjoyed good health. I am confident that I will recover quickly. As long as I follow the doctor's advice and undergo treatment diligently, I am certain to recover faster than others.” (P8)	Previously enjoyed good health	Self-efficacy as a driving force	
	Even minor colds are a rare occurrence		
	Confidence		
	Will have a rapid recovery		
	Will follow the doctor's advice		
	Undergo treatment diligently		
	Certain to recover faster than others		
“Through the process of cultivating healthy lifestyle habits automatically, I have gained a deeper understanding of myself. I am confident in my ability to maintain a regular daily routine and engage in activities that promote physical recovery.” (12)	Cultivate healthy lifestyle habits automatically		
	Gained a deeper understanding of myself		
	Confident in my ability		
	Maintain a regular daily routine		
	Engage in activities that promote physical recovery		

### 2.6 Trustworthiness

Throughout the interview process, the research team conducted regular debriefing sessions to report and discuss challenges encountered during the interviews and subsequently refined the interview protocols and methodologies to enhance the reliability and comprehensiveness of the results. During the data organization and analysis phase, data entry was performed collaboratively by two researchers to minimize the omission of critical information and ensure transcription accuracy relative to the original interview records. Data analysis was likewise conducted jointly by two researchers; in cases of divergent thematic interpretations, discrepancies were resolved through repeated review of the materials or verification with the interviewees, ultimately achieving consensus. Furthermore, the researchers provided timely feedback on the preliminary findings to the participants to ensure that the interpreted themes remained aligned with the original intent of their expressions. Thematic saturation was monitored iteratively throughout the data collection process. After each interview, the research team reviewed the transcripts to assess the emergence of new codes and themes. Data collection concluded after 18 interviews, when no new themes had emerged from the final consecutive interviews, confirming that saturation had been achieved.

### 2.7 Ethical considerations

This research was conducted in accordance with the Declaration of Helsinki and was approved by the Ethics Committee of Sir Run Run Shaw Hospital, School of Medicine, Zhejiang University (registration number: SRRSHEC-2025-0695). Before the interviews began, the research objectives, purpose, and significance of the study were clearly explained to the participants, and the relevant concepts were defined. All participants provided written informed consent. To ensure the privacy of the research participants, all the audio recordings and their corresponding textual transcriptions are securely stored in designated folders exclusively for the analytical purposes of this study. Furthermore, all individual names have been replaced with alphanumeric codes to maintain anonymity.

## 3 Results

Interviews were conducted with 18 older adults with cancer. The participants, aged between 65 and 78 years, had stage I–II cancer and were receiving stable chemotherapy or radiotherapy. The detailed demographic and sociodemographic characteristics of the participants are presented in Table 3.

**Table 3:** Participant characteristics (*N* = 18).

Number	Sex	Age	Marital Status	Type of cancer	Stage	Therapy	Medical insurance	Caregiver
P1	Male	65	Married	Liver cancer	I	Chemotherapy	New rural cooperative medical scheme	Spouse
P2	Male	69	Married	Colorectal cancer	II	Radiotherapy	Urban employee basic medical insurance	Spouse
P3	Male	68	Married	Colorectal cancer	II	Chemotherapy	Urban resident basic medical insurance	Spouse
P4	Female	69	Married	Lung cancer	I	Radiotherapy	New rural cooperative medical scheme	Spouse
P5	Male	68	Married	Colorectal cancer	II	Radiotherapy	Urban resident basic medical insurance	Spouse
P6	Male	78	Married	Colorectal cancer	II	Chemotherapy	New rural cooperative medical scheme	Offspring
P7	Female	68	Married	Cervical cancer	II	Radiotherapy	New rural cooperative medical scheme	Spouse
P8	Female	70	Married	Colorectal cancer	II	Radiotherapy	Urban resident basic medical insurance	Spouse
P9	Female	66	Married	Liver cancer	II	Radiotherapy	Urban resident basic medical insurance	Spouse
P10	Male	68	Married	Colorectal cancer	I	Chemotherapy	New rural cooperative medical scheme	Spouse
P11	Male	67	Married	Colorectal cancer	II	Chemotherapy	Urban employee basic medical insurance	Spouse
P12	Male	73	Married	Liver cancer	II	Radiotherapy	New rural cooperative medical scheme	Spouse
P13	Male	68	Married	Colorectal cancer	II	Chemotherapy	Urban resident basic medical insurance	Spouse
P14	Male	75	Married	Lung cancer	II	Radiotherapy	New rural cooperative medical scheme	Offspring
P15	Female	78	Married	Liver cancer	II	Chemotherapy	New rural cooperative medical scheme	Offspring
P16	Male	67	Married	Colorectal cancer	II	Radiotherapy	New rural cooperative medical scheme	Spouse
P17	Male	67	Married	Colorectal cancer	I	Radiotherapy	New rural cooperative medical scheme	Spouse
P18	Female	76	Married	Colorectal cancer	II	Chemotherapy	Urban resident basic medical insurance	Offspring

**Figure 1:**
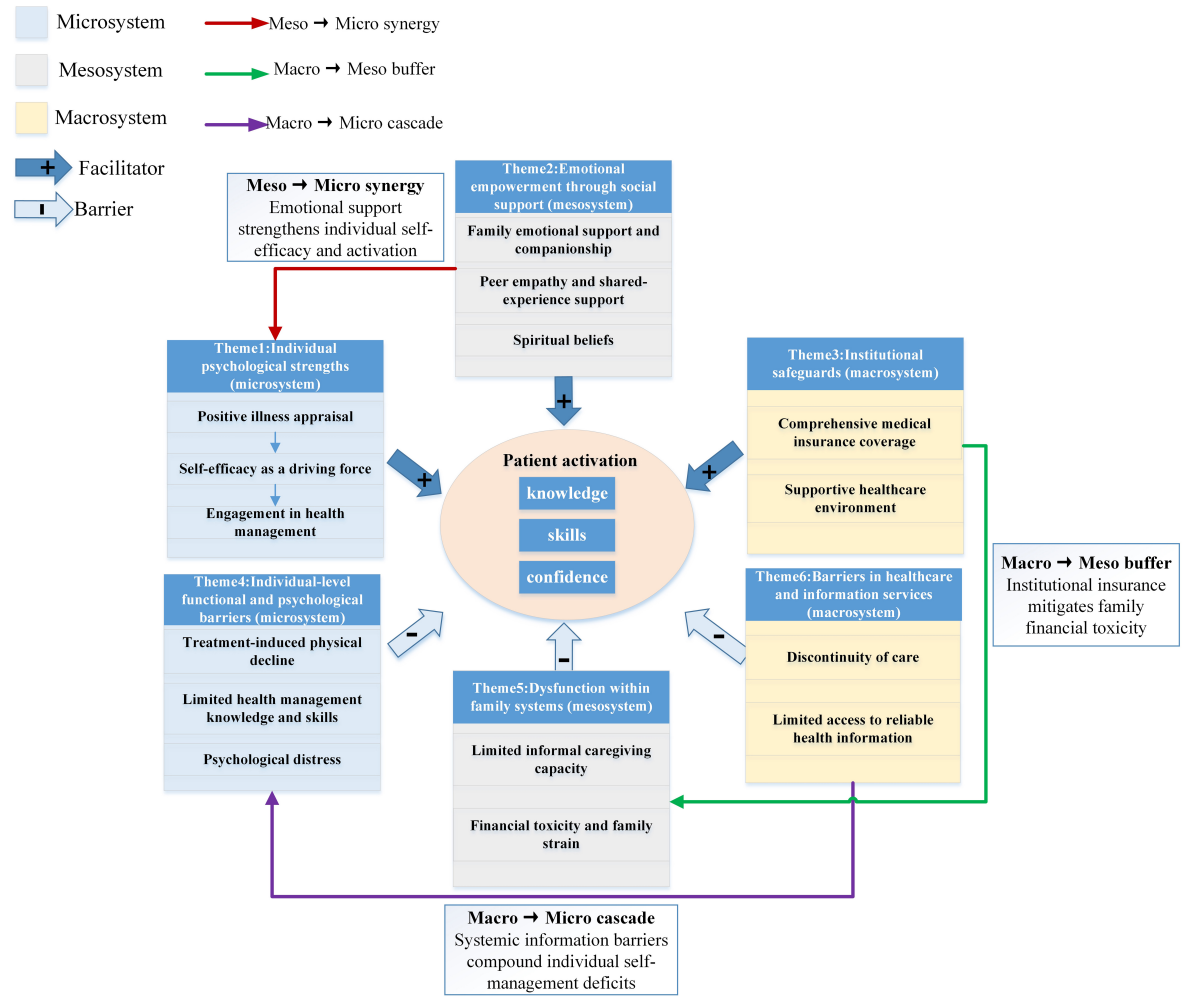
Ecological framework of barriers and facilitators to patient activation among older adults with cancer.

Guided by ecological systems theory, this study identified six themes and 15 subthemes, systematically elucidating the barriers and facilitators influencing patient activation among older adults with cancer (detailed in Table 4). The conceptual framework is illustrated in Figure 1.

**Table 4:** Barriers and facilitators identified among patients based on ecological systems theory.

Category	Theme	Subtheme
Facilitators for patient activation in older adults with cancer	Individual psychological strengths (microsystem)	Positive illness appraisal
		Self-efficacy as a driving force
		Engagement in health management
	Emotional empowerment through social support (mesosystem)	Family emotional support and companionship
		Peer empathy and shared-experience support
		Spiritual beliefs
	Institutional safeguards (macrosystem)	Comprehensive medical insurance coverage
		Supportive health care environment
Barriers for patient activation in older adults with cancer	Individual-level functional and psychological barriers (microsystem)	Treatment-induced physical decline
		Limited health management knowledge and skills
		Psychological distress
	Dysfunction within family systems (mesosystem)	Limited informal caregiving capacity
		Financial toxicity and family strain
	Barriers in health care and information services (macrosystem)	Discontinuity of care
		Limited access to reliable health information

### 3.1 Category 1: facilitators of patient activation in older adults with cancer

#### 3.1.1 Theme 1: individual psychological strengths (microsystem)

- ***Positive illness appraisal***

Most participants reframed their cancer diagnosis within a broader life context, integrating their illness into a positive cognitive narrative rather than perceiving it solely as a threat.

“*At my age, falling ill is natural. Compared to the hardships I endured in my youth, this is just a minor setback. I will calmly undergo treatment without worrying too much.” (P6)*

Some participants actively constructed a narrative of relative fortune by comparing themselves to others they perceived as less fortunate.

“*I have also seen younger people around me lose their lives to cancer or accidents. Compared to them, I am already lucky to have a chance at getting treatment.” (P10)*

- ***Self-efficacy as a driving force***

Beyond cognitive acceptance, participants' confidence in their own resilience further promoted activation. Patients who attributed their prior good health to personal resilience reported stronger confidence in managing treatment and recovery.

“*Previously, I have always enjoyed good health. I am confident that I will recover quickly. As long as I follow the doctor's advice and undergo treatment diligently, I am certain to recover faster than others.” (P8)*

Adopting healthier lifestyle habits reinforced participants' belief in their capacity for self-directed recovery.

“*Through cultivating healthy lifestyle habits, I have gained a deeper understanding of myself. I am confident in my ability to maintain a regular daily routine and engage in activities that promote physical recovery.” (P12)*

- ***Engagement in health management***

This confidence translated into sustained behavioral change as participants embedded health management into their daily routines. Many participants internalized health management as a core component of daily life.

“*Through this experience, my health awareness has significantly improved, and I have come to understand that maintaining healthy lifestyle habits and focusing on disease management are equally important for promoting recovery.” (P1)*

This internalized awareness motivated sustained engagement in health-promoting behaviors.

“*Now, health management has become a self-directed behavior for me, as I feel that participating in it greatly enhances both my physical and mental well-being.” (P7)*

#### 3.1.2 Theme 2: emotional empowerment through social support (mesosystem)

- ***Family emotional support and companionship***

Positive caregiving from family members fostered patients' motivation to engage in self-management and reduced the perceived burden on loved ones.

“*My wife truly works hard. Every day, she prepares diverse and delicious meals for me. I believe that for her and our family, I will keep going with my treatment and try to learn how to take care of myself.” (P3)*

Emotional support from family generated a sense of belonging and hope that sustained patients through treatment.

“*My family is very concerned about my emotions. They chat with me every day, and I don't feel alone with them. I will receive treatment and get better—I really want to be with my family for a long time.” (P9)*

- ***Peer empathy and shared-experience support***

Beyond family ties, fellow patients with cancer offered a distinct form of empathy grounded in shared illness experience rather than preexisting relational bonds. Shared illness experiences created strong emotional connections and reduced feelings of isolation.

“*After communicating with fellow patients, I realized that I was not alone in this battle. I learned from them about psychological adjustment, physical exercise, and nutritional intake, which have been extremely beneficial to my treatment.” (P4)*

Peer support extended beyond face-to-face interactions to online platforms, offering accessible and motivating forms of engagement.

“*Some anticancer live streamers on the internet have a remarkably positive attitude. Moreover, I feel that when many people join me in the recovery process, I am more motivated.” (P11)*

- ***Spiritual beliefs***

For some participants, religious beliefs provided a framework for accepting illness and finding inner peace.

“*When I was first diagnosed with cancer, my world almost collapsed. But with the guidance of Buddhism, I gradually came to accept my fate and stopped complaining and blaming.” (P5)*

#### 3.1.3 Theme 3: institutional safeguards (macrosystem)

- ***Comprehensive medical insurance coverage***

The expansion of medical insurance coverage offered patients a wider range of treatment options and brought them hope for survival.

“*With medical insurance covering part of the medical expenses, I can now try more advanced treatment options without worrying about the cost or missing out on potentially more effective treatments.” (P2)*

Expanded medical insurance coverage broadened treatment options and alleviated financial anxiety.

“*After learning that the reimbursement rate of medical insurance was very high, I felt relieved and decided to proceed with treatment, as it would not impose a heavy financial burden on my family.” (P16)*

- ***Supportive health care environment***

Digital health services, including remote registration and time-slot scheduling, improved convenience for older patients.

“*Previously, securing an appointment with a renowned specialist required queuing overnight. Now, we can select appointment times based on our availability.” (P8)*

### 3.2 Category 2: barriers to patient activation among older adults with cancer

#### 3.2.1 Theme 4: individual-level functional and psychological barriers (microsystem)

- ***Treatment-induced physical decline***

Participants encountered activation barriers across three distinct dimensions—physical, cognitive, and emotional. At the physical level, cancer treatment significantly reduced patients' physical capacity, constraining their ability to maintain active daily routines.

“*Since falling ill, I have noticed a significant decline in my physical strength. I no longer have the physical stamina to engage in the exercises I used to do.” (P10)*

Fatigue, which is commonly reported during chemotherapy and radiotherapy, further limits patients' physical and psychological engagement.

“*After receiving radiotherapy, I have been experiencing an indescribable sense of fatigue. I feel exhausted every day even without doing much. The more exhausted I become, the less I want to move, and the less I move, the more exhausted I feel.” (P17)*

- ***Limited health management knowledge and skills***

At the cognitive level, many participants had a limited understanding of health management beyond following medical instructions, making them uncertain about how to promote their own recovery.

“*I don't really know what health management means. I just know that I follow the doctor's instructions, take my medication on time, and go for regular check-ups.” (P4)*

Comorbidities compounded this challenge, disrupting previously effective self-management routines.

“*Since I found out that I have issues with my blood sugar and blood pressure, I've been very mindful of my diet and lifestyle habits. However, the sudden diagnosis of cancer has completely thrown my previous health plans into disarray.” (P6)*

- ***Psychological distress***

At the emotional level, prevailing beliefs about the harmfulness of chemotherapy generated treatment resistance and fear before initiation.

“*Having heard others talk about chemotherapy and witnessed people around me undergoing it, I noticed that those who receive chemotherapy are all emaciated and listless. To me, chemotherapy is basically equivalent to waiting for death.” (P9)*

Posttreatment symptoms, including persistent fatigue and perceived immune decline, fuelled anxiety about cancer recurrence and metastasis.

“*I feel that my physical condition has deteriorated significantly compared to before. I have a poor appetite and poor sleep quality, and I catch colds easily. I am constantly worried that my weakened immune system might lead to cancer recurrence and metastasis.” (P14)*

#### 3.2.2 Theme 5: dysfunction within family systems (mesosystem)

- ***Limited informal caregiving capacity***

Family-level barriers operated along relational and economic dimensions. In the relational dimension, spouse caregivers' own health limitations and advancing age reduced their capacity to provide adequate care, increasing patients' psychological burden.

“*My spouse's health is also poor. On the one hand, she has to take care of me; on the other hand, she has to look after our grandchild. As she is getting older, she cannot keep up with the demands, and her health is deteriorating. I feel like I am causing them trouble.” (P10)*

Caregivers' limited understanding of the treatment plan and emotional distress transmitted anxiety to patients, undermining their activation.

“*My husband lacks sufficient understanding of my treatment plan and sometimes misinterprets the doctor's instructions. After communicating with the doctor, he becomes extremely anxious even when there are no serious issues, which makes me worried about the effectiveness of my treatment.” (P15)*

- ***Financial toxicity and family strain***

Along the economic dimension, treatment costs generated guilt and family tension, leading some patients to consider abandoning treatment.

“*Every time I see my family struggling to cover my medical expenses, I am deeply concerned that the worsening of my condition will impose an even greater financial burden on my family. This makes it difficult for me to focus on my treatment, and I often contemplate giving up.”* (P6)

Financial strain triggered disagreements among family members over treatment decisions, further destabilizing patients' sense of security.

“*Previously, doctors proposed multiple treatment options. Undoubtedly, more advanced treatment options come with higher costs. The children have already had several arguments over financial matters. I have even considered giving up on treatment.”* (P12)

#### 3.2.3 Theme 6: barriers in health care and information services (macrosystem)

- ***Discontinuity of care***

Weak integration between tertiary hospitals and community-based services left patients without adequate follow-up care after discharge.

“*While the community provides regular health check-ups, the examination items are predominantly basic and unrelated to my current medical condition. After discharge from your facility, I feel as though there is no follow-up care provided.” (P2)*

- ***Limited access to reliable health information***

Brief consultations left patients uncertain about their care and reluctant to raise further questions.

“*The time for communication with physicians is limited. I sometimes have difficulty articulating my concerns effectively, which may result in overlooking pertinent questions and leaving me with lingering uncertainties about the responses I receive.” (P11)*

Online health information was perceived as overwhelming and unreliable, exacerbating rather than alleviating patients' anxiety.

“*When encountering problems, I sometimes do a quick online search. However, the internet bombards me with an overwhelming amount of information. I am uncertain about which category my situation falls into, and there is a plethora of conflicting viewpoints, which only exacerbates my anxiety.” (P18)*

## 4 Discussion

This study identified six themes and 15 sub-themes illustrating how patient activation among older adults with cancer is shaped by factors operating across the microsystem, mesosystem, and macrosystem. Rather than functioning in isolation, these factors appear to interact across ecological levels, producing complex patterns of activation and disengagement. The discussion begins by situating the findings within the Chinese sociocultural context that shapes their interpretation. It then examines the findings through two analytical lenses—intra-individual activation mechanisms and cross-system interactions—before outlining implications for practice and policy. This structure reflects the dual nature of patient activation as an individual-level construct shaped by relational influences from broader ecological levels.

### 4.1 Contextualizing the findings within Chinese sociocultural norms

This section situates the findings within the broader Chinese sociocultural context, drawing on existing literature to interpret patterns that extend beyond the interview data alone. Although participant accounts resonated with several culturally distinctive dynamics, these interpretations are informed primarily by prior research rather than derived directly from thematic analysis. Confucian cultural values emphasize family harmony, filial piety, and respect for authority, often placing collective family interests above individual autonomy in medical decision-making (11, 22). This cultural orientation suggests that older Chinese adults with cancer may not view patient activation as an individual endeavor but rather as a family-centered process in which the patient complies with treatment while the family handles care coordination and emotional support. As two participants explained:

“The children have already had several arguments over financial matters. I have even considered giving up on treatment” *(P12)*, “For my wife and our family, I will keep going with my treatment and try to learn how to take care of myself.” *(P3)*,

illustrating how family dynamics—rather than individual preference—shaped activation-related choices.

Physician paternalism further shapes activation in the Chinese context. A recent cross-cultural comparison between the United Kingdom and China revealed that Chinese patients demonstrated significantly lower participation in medical decision-making and communication, a difference attributable to greater power distance and collectivist cultural norms (23). However, such differences may manifest as apparent passivity such that Western-oriented activation measures could be misinterpreted as low motivation when they in fact reflect culturally sanctioned respect for medical expertise. The digital divide among older Chinese adults adds further complexity (24, 25). Although telemedicine and online health platforms facilitated activation for some participants in this study, many older adults lacked the digital literacy to navigate these resources independently, creating a paradox in which institutional advances in health care delivery may inadvertently exclude the populations most in need.

Collectively, these cultural and structural features suggest that patient activation models developed in Western contexts, which focuses on individual autonomy and self-directed health management, require adaptation for Chinese older adults with cancer.

### 4.2 Individual-level activation: pathway and barriers

The findings suggest that patient activation among older adults with cancer follows a progressive pathway from cognitive reframing to behavioral engagement, with self-efficacy described by participants as a connecting resource. Participants who reframed their cancer diagnosis within a broader life context, often drawing on Confucian cultural narratives of acceptance and maturity, demonstrated greater readiness to engage in health management. This cognitive reframing appeared to support self-efficacy, which in turn motivated the integration of health management into daily routines. This progression aligns with the four-stage developmental model underlying the Patient Activation Measure, in which individuals advance from recognizing the importance of an active role in their care, to building knowledge and confidence, and ultimately to maintaining health behaviors under stress (26). Among cancer patients specifically, this multidimensional progression manifests in core components including information seeking, participation in treatment decision making, healthcare confidence, and self-management behaviors (27).

However, three interrelated barriers at the individual level can disrupt this pathway. Physical decline resulting from cancer treatment creates a self-reinforcing cycle: fatigue reduces activity, which further diminishes physical capacity and erodes motivation (28). Insufficient health management knowledge compounds this problem—when patients conflate treatment side effects with disease process, their anxiety intensifies and willingness to engage declines. Although this mechanism has been most clearly documented in other chronic disease populations such as type 2 diabetes (29), the pattern observed in the present study suggests it extends to the cancer context. Negative emotions, particularly the fear of cancer recurrence, further undermine activation by redirecting psychological resources away from self-management toward worry (30). Notably, these three barriers—physical, cognitive, and emotional—appear to cooccur rather than operate independently. Participants who described treatment-induced fatigue simultaneously expressed confusion about whether their symptoms reflected normal treatment effects or disease progression, and this uncertainty fuelled recurrent anxiety. Such clustering suggests that single-dimensional interventions may be insufficient. Effective activation interventions therefore appear to require simultaneous attention to multiple barrier dimensions.

### 4.3 Cross-system interactions: three pathways extending EST

Notably, the barriers to and facilitators of patient activation appear to extend beyond isolated ecological levels, with participants' accounts suggesting interconnections among the microsystem, mesosystem, and macrosystem. This observation is consistent with frameworks proposed for understanding influences across the cancer care continuum, which conceptualize policy, institutional, interpersonal, and individual factors as interconnected rather than independent layers (31). Drawing on participants' narratives, three patterns of cross-level interaction can be discerned.

First, mesosystem emotional support appears to facilitate microsystem activation. Family companionship and encouragement were described by participants not only as sources of emotional comfort but also as reinforcing their cognitive acceptance of illness and strengthening their self-efficacy for health management. Participants who described their family as emotionally supportive reported greater confidence and more proactive health behaviors; peer support from fellow patients complemented this by offering distinct forms of empathy rooted in shared illness experience, particularly regarding symptom interpretation and treatment expectations—together suggesting that interpersonal resources at the mesosystem level may help cultivate individual-level activation. Quantitative research in related populations provides a convergent context for this observation: social support has been shown to influence health-promoting behaviors with patient activation playing a partial mediating role (32), and among older Chinese adults with chronic conditions, illness acceptance has been identified as a significant mediator between social support and self-management activation (33). These quantitative studies cannot confirm our qualitative findings, but the parallel patterns observed across different populations suggest that this connection between emotional support and individual activation is worth investigating further.

Second, macrosystem institutional safeguards appear to buffer mesosystem vulnerability. Comprehensive medical insurance coverage functioned as a structural facilitator whose benefits participants described as extending across ecological levels. At the mesosystem level, insurance was reported to reduce family financial strain, helping to prevent the guilt and intrafamily conflict that might otherwise undermine patients' activation (34, 35). At the microsystem level, participants who felt financially protected reported greater willingness to pursue treatment and engage in health-promoting behaviors. This pattern suggests that macrolevel policy interventions may contribute to downstream activation benefits that extend beyond their immediate financial function, although the mechanisms through which this occurs require further examination.

Third, macrosystem barriers may compound microsystem deficits. Participants who experienced fragmented care coordination between tertiary hospitals and community services described difficulty accessing reliable health information, which they associated with heightened knowledge gaps and emotional distress at the individual level (36). Similarly, brief physician consultations are a structural feature of China's high-volume tertiary hospital system. These consultations were reported to leave patients with unanswered questions and unaddressed concerns (37), potentially intensifying the negative emotions and self-management confusion described at the microsystem level. This pattern suggests that individual-level barriers cannot be fully understood or effectively addressed without considering the institutional conditions that shape them.

### 4.4 Implications for practice and policy

The multilevel and interactive nature of patient activation observed in this study has several implications for clinical practice and health policy.

At the clinical level, oncology nurses should assess activation through a multidimensional lens that examines physical capacity, health literacy, and emotional well-being concurrently. Brief validated tools such as the Patient Activation Measure could be administered at diagnosis and at key treatment transitions to identify which barrier dimension most constrains each patient's activation. Structured exercise programs tailored to patients' physical capacity levels, combined with treatment side-effect education, could address the clustering of physical, cognitive, and emotional barriers within a single integrated intervention (28).

At the family level, interventions should target the patient–caregiver dyad rather than the patient alone. Given the centrality of family in Chinese cancer care, and evidence that caregiving stress predicts long-term physical impairment in caregivers (38), caregiver training programs addressing disease management, emotional support strategies, and self–care strategies to prevent caregiver burnout could strengthen the mesosystem resources that support individual activation (39, 40). We have identified three key priorities at the institutional and policy levels. First, strengthening the integration between tertiary hospitals and community-based primary care services would address the care fragmentation identified as a key macrosystem barrier. A recent study of older patients with cancer under China's hierarchical diagnosis and treatment system confirmed that patients receive “no guidance for connecting with different levels of doctors” and face an “absence of integrated medical records” between hospitals and primary care facilities (41). Establishing community oncology rehabilitation programs staffed by trained nurses, in conjunction with integrated care models advocated for older cancer populations (42), could provide the continuity of care that patients currently lack after hospital discharge. Second, health care providers should integrate peer support networks into routine oncology care. These networks, which include face-to-face cancer survivor groups and curated digital platforms, already exist naturally outside formal health care systems and represent an untapped source of patient activation (43, 44). Third, the reimbursement scope of basic medical insurance for older cancer patients should be expanded to include outpatient rehabilitation services and supportive care medications, thereby reducing family financial strain that undermines patient activation. National cancer control programs should also require the integration of oncology rehabilitation into primary care service packages, with dedicated funding for community-based cancer survivorship programs.

### 4.5 Limitations

This study has several limitations. First, all participants were recruited from a single tertiary hospital in an urban setting, which limits the transferability of the findings to rural or community-based contexts where health care resources and cultural dynamics may differ substantially. Second, the sample comprised only patients with stage I–II cancer who were receiving stable treatment; the experiences of those with advanced-stage disease, active treatment complications, or palliative care needs were not captured and may involve qualitatively different activation patterns. Third, purposive sampling may have introduced selection bias, as participants willing to be interviewed may represent a more activated subset of the population. Fourth, the cross-sectional design provides a snapshot of activation at one point in time but cannot capture how activation evolves across treatment phases. Longitudinal and multisite qualitative studies are needed to address these gaps.

## 5 Conclusion

In this study, ecological systems theory was applied to patient activation among older adults with cancer, and three cross-system interaction pathways were identified. Family and peer emotional support appeared to amplify individual activation, institutional safeguards buffered family-level vulnerability, and care fragmentation compounded individual barriers. The findings also suggest that Confucian cultural values shape activation as a family-centered process and that Western-derived activation models require cultural adaptation. Longitudinal and multisite studies are needed to examine how activation evolves across treatment phases. Multilevel interventions targeting individual capabilities, family resources, and institutional care continuity should also be evaluated.

## Data Availability

The original contributions presented in the study are included in the article/supplementary material, further inquiries can be directed to the corresponding author.
